# Expression of Concern: Study of the biological function of LncRNA LUCAT1 on cervical cancer cells by targeting miR-199b-5p

**DOI:** 10.1042/BSR-20200422_EOC

**Published:** 2020-07-24

**Keywords:** cervical cancer, diagnosis, invasion, LUCAT1, miR-199b-5p, proliferation

The Editorial Office has been made aware of potential issues surrounding the scientific validity of this paper, hence has issued an expression of concern to notify readers whilst the Editorial Office investigates.

